# Sex-Specific Difference in Outcomes after Transcatheter Mitral Valve Repair with MitraClip Implantation: A Systematic Review and Meta-Analysis

**DOI:** 10.1155/2022/5488654

**Published:** 2022-02-21

**Authors:** Fuqiang Sun, Honghao Liu, Qi Zhang, Jiawei Zhou, Haibo Zhan, Fanfan Lu

**Affiliations:** ^1^Department of Cardiovascular Surgery, the First Affiliated Hospital of Zhengzhou University, Zhengzhou, China; ^2^Department of Emergency, the First Affiliated Hospital of Zhengzhou University, Zhengzhou, China; ^3^Department of Endovascular Surgery, the First Affiliated Hospital of Zhengzhou University, Zhengzhou, China

## Abstract

**Background:**

Implantation of the MitraClip is a safe and effective therapy for mitral valve repair in patients ineligible for surgery or at high risk of adverse surgical outcomes. However, only limited information is available concerning sex differences in transcatheter mitral valve repair. We therefore sought to conduct a comprehensive meta-analysis of studies that investigated differences between men and women in outcomes following MitraClip implantation.

**Methods:**

The PubMed and Embase databases were searched until November 2019 for studies reporting outcomes after MitraClip implantation in women versus men. Outcomes included all-cause mortality and major complications at 30 days and one year of follow-up.

**Results:**

Six studies (*n* = 1,109 women; *n* = 1,743 men) were analyzed. At 30 days, women had a similar risk of postoperative complications, such as stroke, major bleeding, and pericardium effusion, without differences in all-cause mortality, procedure success, or MitraClip usage. At one year, the all-cause mortality, the reduction of mitral regurgitation, and the risk of rehospitalization for heart failure were also comparable between male and female patients.

**Conclusion:**

Gender disparity was not found in complications or prognosis of patients undergoing MitraClip implantation. This study suggests that gender should not be considered as a critical factor in the selection of patients as candidates for MitraClip implantation of concern during follow-up.

## 1. Introduction

Mitral regurgitation (MR), the most prevalent form of valvular heart disease, affects nearly 10% of people older than 75 years of age [1]. The mortality rate in such patients reaches 50% at five years of follow-up, and up to 90% of surviving patients experienced at least one hospitalization for heart failure within five years after the diagnosis of severe MR [2]. Mitral valve (MV) repair or replacement is the gold-standard treatment for MR, but some studies have reported that ∼50% of patients with severe symptomatic MR were denied surgical interventions mostly due to advanced age, impaired left ventricular function, and a high comorbidity burden [2,3]. Percutaneous edge-to-edge MV repair with MitraClip (MC) is based on the surgical technique first described by Alfieri [4] and, currently, is the only guidelines-recommended transcatheter treatment available for managing primary or secondary MR [5].

In the vast majority of cardiovascular diseases, there are well-described differences between women and men in terms of epidemiology, pathophysiology, clinical manifestations, the effects of therapy, and outcomes [6]. Gender disparity in cardiac diagnosis and treatment has been investigated thoroughly since Ayanian first described this phenomenon in 1991 [7]. In patients who underwent coronary artery bypass grafting (CABG), it was found that female sex persists as an independent risk factor for adverse outcomes after CABG [8]. In patients undergoing MV surgery, female gender has been identified as being associated with more significantly impaired postoperative long-term survival relative to men [9]. The current evidence with regard to the impact of sex on outcomes in MC implantation is insufficient and conflicting; thus, given this remains an overall unresolved and poorly described issue that has significant implications with regard to patient selection for this procedure, we therefore conducted the present meta-analysis of limited studies focused on directly comparing women and men who received this device in terms of short-term outcomes and mortality during follow-up.

## 2. Methods

### 2.1. Study Objective and Search Strategy

The primary aim of this meta-analysis was to evaluate the influence of sex on clinical outcomes in high-risk patients undergoing MC implantation.

**Figure 1:**
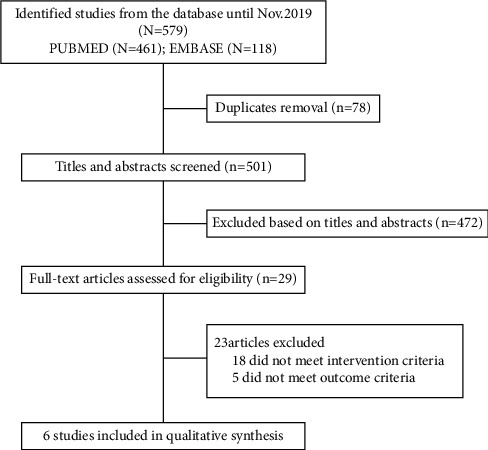
Flowchart of meta-analysis.

The present research was conducted according to the current guidelines, including the recent Preferred Reporting Items for Systematic Reviews and Meta-analyses amendment to the quality of reporting of meta-analyses statement and recommendation from the Cochrane Collaboration and the Meta-analysis of Observational Studies in Epidemiology [10,11]. We searched the PubMed and Embase databases from inception until November 2019, without language restrictions, using the keywords “sex,” “gender,” “men,” “women,” “male,” “female,” “edge-to-edge,” “mitral clip,” “TMVr,” and “transcatheter mitral valve repair” both separately and in combination with one another. We restricted our analysis to published data. References from reviews and selected reports were also examined for additional potentially relevant citations. Eligible studies were selected by two independent reviewers (SFQ and LHH).  Figure 1 outlines the search strategy and pathway followed to gather the final included studies.

### 2.2. Study Identification and Extraction

We performed text searches for studies that met the following criteria: (1) examined clinical outcomes in patients with MC implantation, (2) included a direct comparison between males and females, (3) provided enough information to calculate the effect sizes, and (4) had available patient baseline data. Only original articles were considered for this meta-analysis; case reports, case series, and conference abstracts without a complementary peer-reviewed manuscript publication were not included. In the case of duplicate reporting, the manuscript with the largest patient sample size was selected.

SFQ and ZQ extracted all the data independently, and discrepancies were resolved by consensus. The following information was collected: (1) first author's name, (2) year of publication or presentation, (3) total size and subgroup sample size for both men and women, (4) demographic information, (5) period of follow-up, (6) data of postoperative adverse events, (7) procedural characteristics, (8) 30-days/in-hospital all-cause mortality, and (9) survival curve.

### 2.3. Endpoints

The primary efficacy endpoint was mortality from any cause at 30 days and one year of follow-up. The secondary endpoints were as follows: (1) in-hospital clinical outcomes such as stroke, major bleeding, and pericardium effusion; (2) procedural characteristics such as procedure failure and number of MCs implanted; and (3) rehospitalization and MR reduction at one year of follow-up.

### 2.4. Statistical Analysis

Continuous variables are reported as means and standard deviations, while categorical variables are expressed as numbers (%). The effects of gender on the outcomes of the MC procedure were presented as risk ratios (RRs) and mean differences (MDs) with 95% confidence intervals (CI), using a fixed-effects model. Alternatively, random-effects meta-analyses were performed when between-study variability existed. The hazard ratio (HR) and its 95% confidential interval (95% CI) were used to delineate the effect size for survival. When Kaplan−Meier curves were provided instead of HR values, two researchers independently estimated the HRs indirectly from the curves using Engauger Digitizer version 9.0 according to the methods described by Tierney and others. [12]. Heterogeneity was quantified using Cochran's Q-statistic and I^2^ index tests, and a result of 25% was considered to indicate significant heterogeneity. Sensitivity analysis was conducted by removing low-quality studies and interchanging calculation models (fixed-effects and random-effects) in order to observe outcome stability. Publication bias was assessed using the visual inspection of funnel plots and by Egger's and Begg's regression and was considered significant if found to be present in all tests. All analyses were conducted using Stata 14.0 (StataCorp LLC, College Station, TX, USA), and statistical significance was indicated by *p* < 0.05.

## 3. Results

### 3.1. Baseline Characteristics

The flowchart of study selection is shown in Figure 1. Our electronic search initially yielded 579 citations that were evaluated for eligibility at the title and abstract levels. Once duplicate and irrelevant publications were removed, the full texts of six reports incorporating a total of 1,109 women and 1,743 men were evaluated further for eligibility and were finally included in the current meta-analysis [13–18]. All of the included studies had good methodological quality, indicating a low risk of bias, and were classified as being of high quality based on the Newcastle–Ottawa Assessment scale using nine different parameters (Table 1). The baseline characteristics of the patients in different studies are summarized in Table 2. Among male patients, the mean age ranged from 70.3 to 74.4 years, while the mean age among female patients ranged from 72.8 to 78.9 years.

### 3.2. Short-Term Mortality

**Figure 2:**
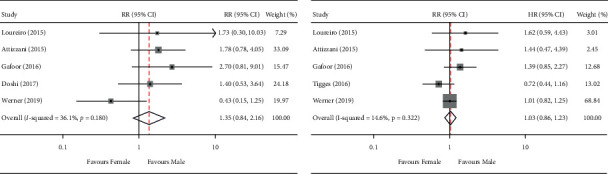
Male versus female. (a) Risk ratio with 95% CI for the composite endpoint of in-hospital mortality; (b) hazard ratio with 95% CI for the composite endpoint of long-term survival.

All six studies reported sex-specific crude mortality rates at 30 days; however, in the study by Tigges and others, there was no death during hospitalization and, thus, it was excluded in the final pooled analysis. A total of 22 death occurred in 879 women and 29 events occurred in 1,381 men. There was no significant difference in the all-cause mortality rate at 30 days between male and female patients (pooled RR: 1.35, 95% CI: 0.84–2.16; I^2^ = 36.1%; *p*=0.18) (Figure 2(a)). Analysis conducted using a random-effects model yielded similar results. Further, the results remained stable when omitting individual studies (Figure S1(a)). No significant publication bias was observed, affirmed by visual inspection of the funnel plots (Begg's test statistic: *p*=1; Egger's test statistic: *p*=0.952) (Figure S2(a)).

### 3.3. One-Year Mortality

The data of long-term survival were derived from studies with a follow-up of one year. As shown in Figure 2(b), five studies provided survival information, and the merged outcomes indicated that all-cause mortality at one year was similar in both women and men (pooled HR: 1.03, 95% CI: 0.86–1.23; I^2^ = 14.6%; *p*=0.32). Similar results were obtained using a random-effects model. Sensitivity analysis was performed by excluding individual studies, and there was no significant change noted in the overall results (Figure S1(b)). No significant publication bias was observed (Begg's test statistic: *p*=0.81; Egger's test statistic: *p*=0.51) (Figure S2(b)).

### 3.4. Stroke

**Figure 3:**
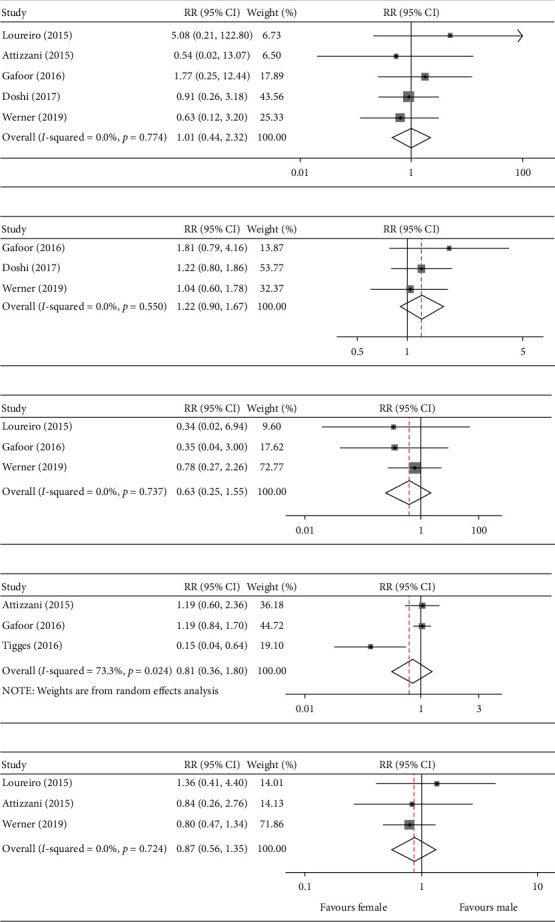
Male versus female. Risk ratio and 95% CI for the pooled endpoints of stroke (a), major bleeding (b), pericardium effusion (c), MR reduction (d), and rehospitalization (e).

With regard to the effect of gender on in-hospital stroke, there were nine events recorded among 849 women and 14 events recorded among 1,338 men in five trials. After meta-analysis, we found that the incidence of stroke among male patients was similar to that among female patients (pooled RR: 1.01, 95% CI: 0.44–2.32; I^2^ = 0.0%; *p* = 0.77) (Figure 3(a)).

### 3.5. Major Bleeding and Pericardium Effusion

Three studies provided data on major bleeding during hospitalization. As shown in Figure 3(b), there was no significant gender difference: 93 out of 731 women developed bleeding after MC implantation, while 98 out of 940 men presented the same (pooled RR: 1.22, 95% CI: 0.90–1.67; I^2^ = 0.0%; *p* = 0.55). We also identified three studies that contained the data on pericardium effusion; however, no gender difference was found when comparing between male and female patients (pooled RR: 0.63, 95% CI: 0.25–1.55; I^2^ = 0.0%; *p* = 0.74) (Figure 3(c)).

### 3.6. MR Reduction and Rehospitalization at One Year of Follow-Up

We compared the rates of patients with MR of less than grade 2 at one year of follow-up to detect the difference in the MR reduction by MC implantation between men and women. The pooled results showed that there was no significant difference in MR reduction between male and female patients undergoing MC therapy (pooled RR: 0.81, 95% CI: 0.36–1.80; I^2^ = 73.3%; *p* = 0.02) (Figure 3(d)). The random-effect size was used for the indication for moderate heterogeneity. Similar corrections were also identified in the comparison of the rates of rehospitalization at one year post procedure; female sex showed no association with an increased risk of rehospitalization relative to men pooled (RR: 0.87, 95% CI: 0.56–1.35; I^2^ = 0.0%; *p* = 0.72) (Figure 3(e)).

### 3.7. Procedure Failure and Number of Clips Implanted

**Figure 4:**
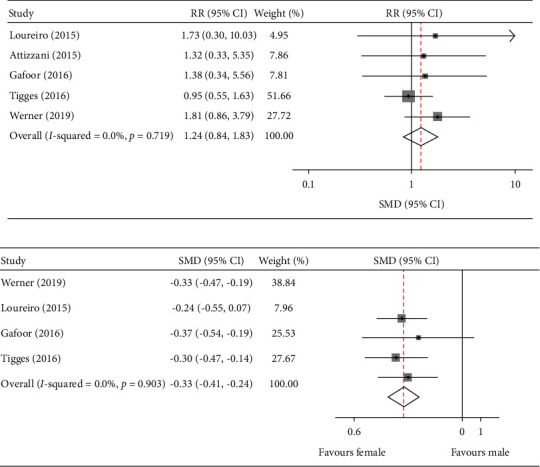
Male versus female. (a) Risk ratio and 95% CI for the comparison of procedure failure. (b) MD and 95%CI for the pooled endpoint of number of clips implanted.

The difference in acute procedure success was assessed between female and male patients after MC implantation. Ultimately, we observed a similar rate of procedure failure, indicating that there was no sex difference in the rate of acute procedure success between men and women pooled (RR: 1.24, 95% CI: 0.84–1.83; I^2^ = 0.0%; *p* = 0.72) (Figure 4(a)). However, we found that female sex was associated with a trend toward less MCs being implanted as compared with male patients (pooled SMD: −0.33, 95% CI: −0.41 to −0.24; I^2^ = 0.0%, *p* = 0.90) (Figure 4(b)).

## 4. Discussion

To the best of our knowledge, this is the first meta-analysis to assess gender-related differences in clinical outcomes following transcatheter MV repair. For decades, we have known that including “female” in surgical risk models used for clinical decision-making dramatically raises the predicted risk in MV surgery [19]; however, there are limited data available for assessing sex disparities with clinical outcomes in patients undergoing treatment with MC, which is the only percutaneous technology currently approved by the Food and Drug Administration for MV repair in the United States. The major findings of the present meta-analysis are as follows: (1) MC implantation revealed high safety and efficacy results in both male and female patients, leading to a considerable reduction in MR grade postprocedure; (2) male and female patients treated by MC implantation had similar mortality rates at 30 days and one year of follow-up, respectively; (3) no significant differences were identified between male and female patients with respect to postoperative major complications; and (4) female gender seems to be associated with a slightly longer procedure time and hospital stay relative to male gender.

Published data on gender-specific survival in MC are scarce. Both studies by Tigges and others and Werner and others were included in our final research, despite few data being collected from Tigges's single-center result in the TRAMI registry including 1064 patients enrolled at 21 different German sites. The overlapped cases were few and most of the measurement outcomes were different in the two studies. Beyond that, the deletion of Tigges's study did not change the pooled endpoint comparisons of long-term mortality, procedure failure, and number of clips implanted in our study. Importantly, the current study does not support the hypothesis that female sex is a risk factor for MC-related mortality. This is underlined by the well-documented fact that greater operative mortality and worse survival following MV surgery in women than in men is due to the intrinsic higher gender-specific morbidity and mortality risks after surgery [20,21]. Women who undergo MV surgery are usually older and carry a higher preoperative risk when compared with men; in addition, the higher rates of MV replacement compared with repair in females could help to explain the worse outcomes observed [9,22]. The lack of MV repair-related benefits including improved short-and long-term survival may be taken into account for the comparability of data following surgical versus transcatheter approaches. The disparity may also be explained partially by the fact that patients undergoing MC are at prohibitive surgical risk and thus oftentimes considered inoperable. Additionally, as MC remains a relatively novel device with its own learning curve, this may have impacted the outcomes of acute mortality in our analysis. Smaller stroke volumes in women than in men have been found in otherwise healthy individuals; however, women with atrial fibrillation have a higher risk of stroke than men [23]. Our study adds clarity to the current literature, which has been inconsistent in terms of sex-related differences after MC implantation. We found a similar stroke risk at 30 days between the two genders; however, this result should be carefully considered due to the unclear potential explication. Eggebrecht and others previously reported bleeding to be among the most frequent major adverse events after MC that are associated with increased in-hospital mortality [24], while elsewhere, the major bleeding rate was almost doubled and significantly higher in women than in men in the TRAMI registry [18]. However, in this investigation, we failed to find significant gender-related differences in terms of the risk of bleeding postprocedure; meanwhile, a similar trend was seen toward the risk of pericardium effusion, which can partially be explained by the advanced age in women but worse baseline vascular comorbidities in men. The underlying results emphasize the need for thorough clinical monitoring for vascular and bleeding complications in both female and male patients after MC implantation. Despite the fact that women tend to have anterior or bileaflet prolapse and MV calcification and men more frequently have posterior leaflet prolapse [25], we still identified that acute procedure success was high and a remarkable reduction in MR was mostly sustained and presented in both groups at one year, indicating that the benefit of MC implantation was obvious in both genders, even in patients at prohibitive risk. In addition, the number of MCs used in the two groups was not significantly different, and thus, we considered that the variations in anatomy may not affect the final MC location. Rates of rehospitalization for heart failure were relatively high in most studies included in our analysis, but no significant differences were seen between men and women, reflecting that this result was not driven by gender-specified differences.

### 4.1. Limitations

There are some limitations to the current meta-analysis as well as in the included publications. First, the main limitation is the inclusion of observational data from studies and registries, subjecting our analysis to the possible bias. Second, the reported outcomes were unadjusted, and the HRs for long-term mortality were derived from the survival curve, so the strength of evidence for pooled outcomes should therefore be interpreted with caution. Third, most patients were included based on receiving MC therapy rather than based on specific indications or anatomic criteria; in addition, details about the prespecified medical therapy strategy and MR etiology were absent, and thus, these may be confounding variables that affected the outcomes in this study. Finally, the power of the Egger's and Begg's tests for funnel plot asymmetry is too low to distinguish chance from real asymmetry, given there were no more than 10 studies eligible for the pooled analysis; thus, the possibility of potential publication bias cannot be ruled out.

## 5. Conclusion

In the present meta-analysis involving 2,852 patients, there were no major gender-specific differences in complications and prognosis after MC implantation. This study suggests that gender should not be considered as a critical factor in the selection of patients as candidates for MC or a concern during follow-up, and we hope that our results will assist in such decision-making. Further large-scale randomized trials are recommended to better explore these results.

## Figures and Tables

**Table 1:** Characteristics of included MC studies.

Study	Year	Study design	Location	Patients			Doe	Follow-up	NOS
				All (*n*)	Women (%)	Men (%)			
Werner	2019	Multicenter (P)	Germany	828	39.5	60.5	2010–2013	1 year	7
Doshi	2017	Multicenter (R)	America	521	42.0	58.0	2012–2014	30-days	5
Gafoor	2016	Multicenter (P)	Europe	567	36.2	63.8	2008–2011	1 year	7
Tigges	2016	Single-center(R)	Germany	592	38.9	61.1	2008–2015	2.13 years (M)	5
Loureiro	2015	Multicenter(R)	Europe	173	37.0	63.0	2009–2012	16 months	6
Attizzani	2015	Single-center(P)	Italy	171	38.0	62.0	2008–2013	1 year	6

Doe: date of enrollment; M: mean; P: prospective; R: retrospective; NOS : Newcastle–Ottawa Scale.

**Table 2:** Baseline characteristics of patients.

Study	Age		LES		Diabetes		Hypertension		COPD		Prior Stroke		AF		FMR		NYHA III/IV		Prior MI (%)	
	(years)		(%)		(%)		(%)		(%)		(%)		(%)		(%)		(%)			
	F	M	F	M	F	M	F	M	F	M	F	M	F	M	F	M	F	M	F	M
Werner	77	74.2	24.2	24.7	34.8	29.0	77.2	78.5	20.8	24.7	7.4	12.0	47.8	41.8	67.6	71.8	90.2	88.3	21.9	31.2
Attizzani	74.0	70.3	8.0#	7.7#	36.9	34.0	73.8	74.5	21.5	21.7	9.2	7.5	33.8	42.5	75.4	82.1	83	80.2	20	42.5
Gafoor	76	72	23.6	22.7	--	--	--	--	13.9	21.9	5	7	67	68	33.2	22.7	87.4	83.5	21	38
Tigges	76.0	74.4	19.5	22.9	19.9	32.9	74.2	70.4	18.0	21.9	12.3	18.3	64.9	69.3	--	--	94.7	96.1	21.8	40.4
Loureiro	78.9	73.5	19.4	18.3	11	26	64	61	27	14	5	11	44	46	48	58	97	95	20	46
Doshi	72.8	73.3	--	--	19.2	23.8	64.8	68.5	21.5	25.2	--	--	--	--	--	--	--	--	12.3	13.2

^#^: Logistic EuroSCORE II; LES : logistic EuroSCORE; AF : atrial fibrillation; FMR : functional mitral regurgitation.

## Data Availability

All the data are included within the article.

## Supplementary Materials

Supplementary Materials Figure S1 : sensitivity analysis for in-hospital mortality (a) and long-term mortality (b); Figure S2 : publication bias analysis for in-hospital mortality (a) and long-term mortality (b). .
