# Supplementary figures and images for: Sex-Specific Difference in Outcomes after Transcatheter Mitral Valve Repair with MitraClip Implantation: A Systematic Review and Meta-Analysis
Source: J Interv Cardiol. 2022 Feb 21;2022:5488654. doi: 10.1155/2022/5488654 (PMC8885186; doi:10.1155/2022/5488654)

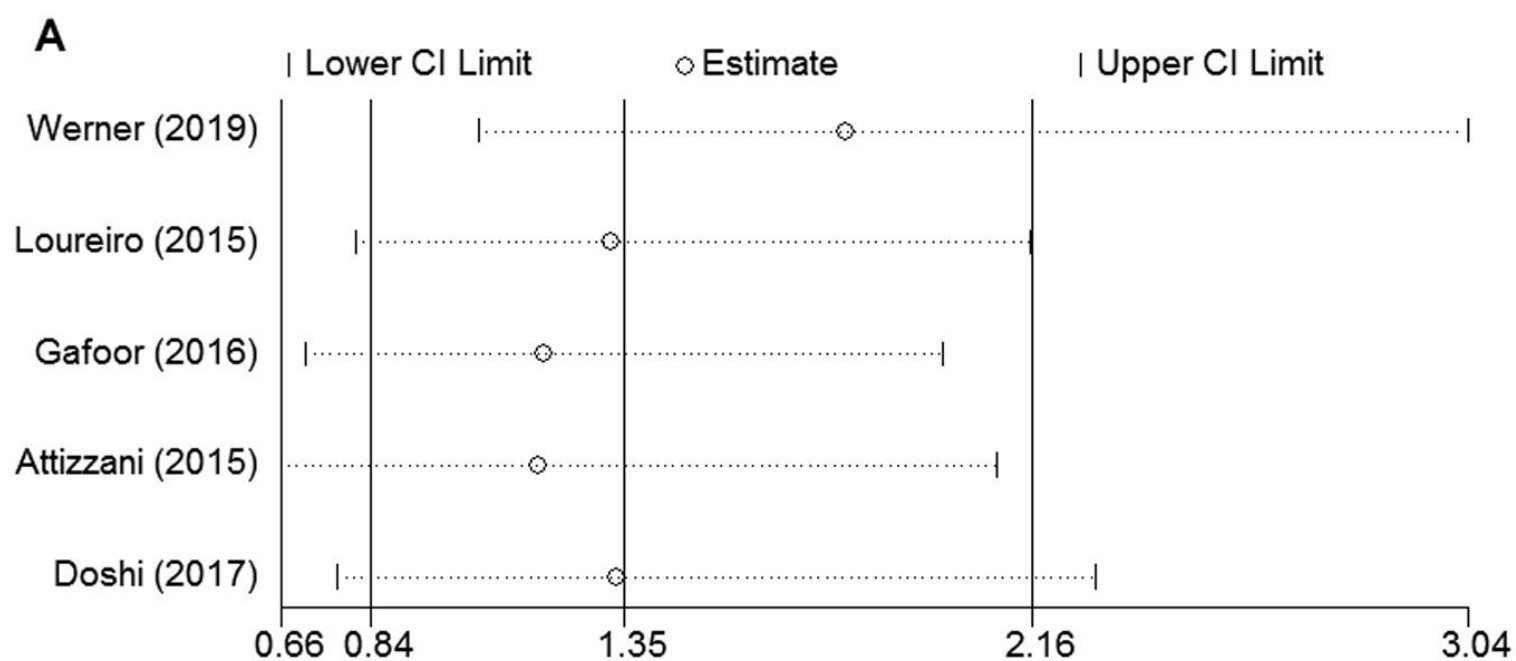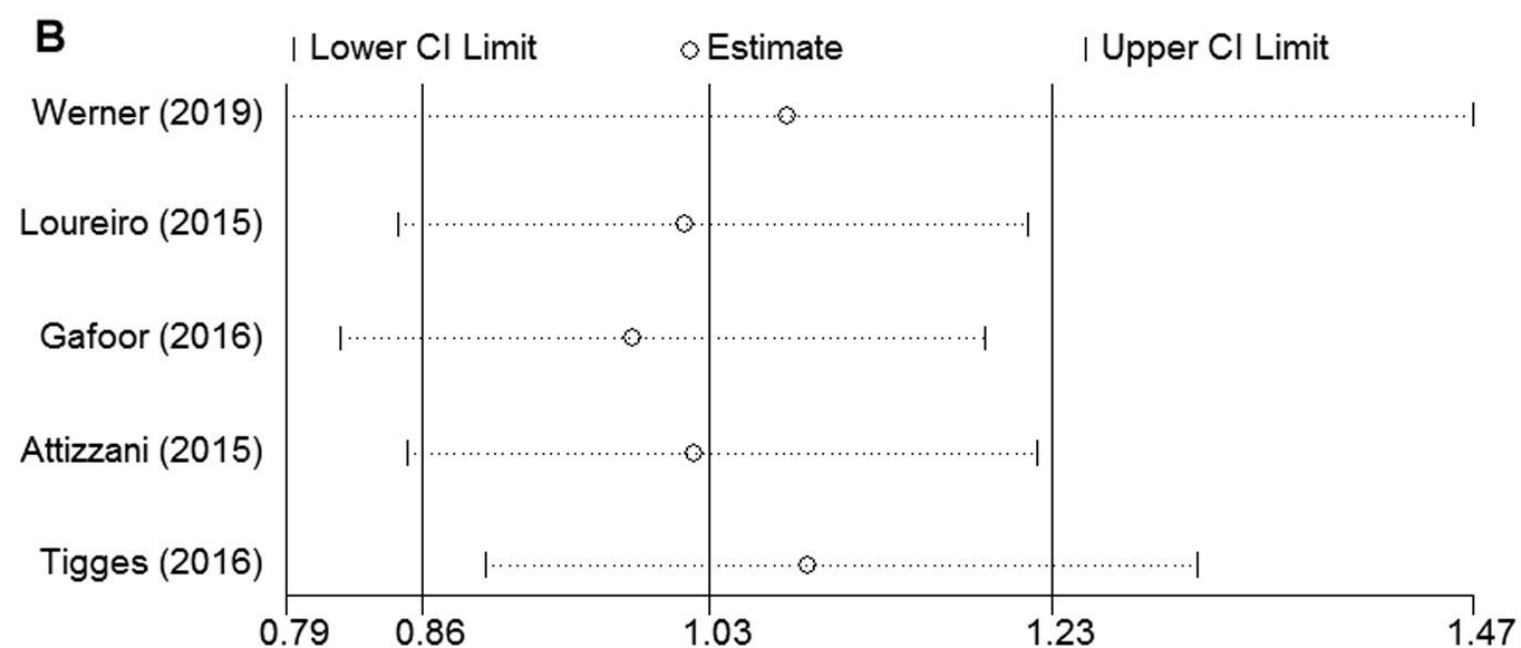

Supplement: Supplementary Materials — Figure S1 : sensitivity analysis for in-hospital mortality (a) and long-term mortality (b); Figure S2 : publication bias analysis for in-hospital mortality (a) and long-term mortality (b). . [file 5488654.f1.zip › 5488654.f1/figure_S1.pdf]

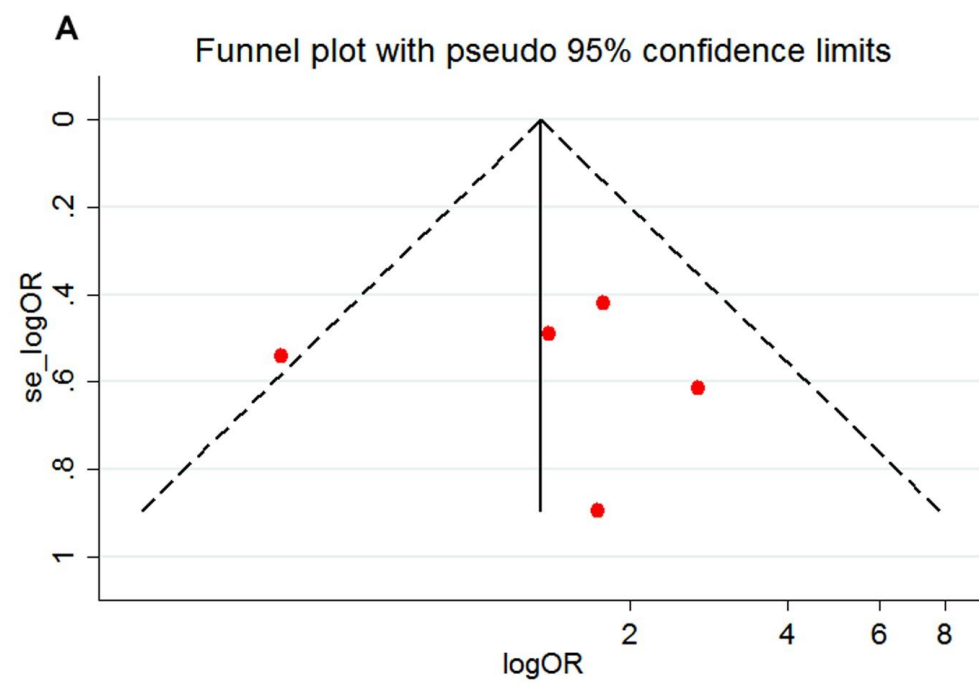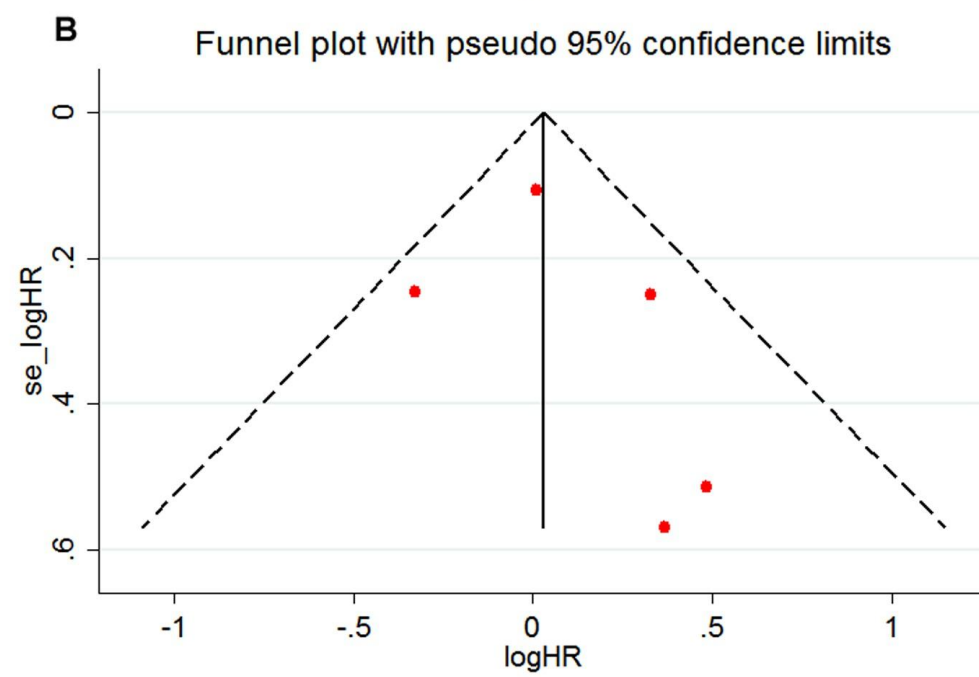

Supplement: Supplementary Materials — Figure S1 : sensitivity analysis for in-hospital mortality (a) and long-term mortality (b); Figure S2 : publication bias analysis for in-hospital mortality (a) and long-term mortality (b). . [file 5488654.f1.zip › 5488654.f1/figure_S2.pdf]
